# Supplementary material for: Intercavity polariton slows down dynamics in strongly coupled cavities
Source: Nat Commun. 2024 Apr 4;15:2915. doi: 10.1038/s41467-024-47336-y (PMC10994920; doi:10.1038/s41467-024-47336-y)
Supplement: Supplementary file 1 — Supplementary Information [file 41467_2024_47336_MOESM1_ESM.pdf]

Supplementary Information

Yesenia A. García Jomaso,[1] Brenda Vargas,[1] David Ley Domínguez,[1] Román J. Armenta-Rico,[1] Huziel E. Sauceda,[1] César L. Ordoñez-Romero,[1] Hugo A. Lara-García,[1] Arturo Camacho-Guardian,[1, *] and Giuseppe Pirruccio[1, †]

[1] *Instituto de Física, Universidad Nacional Autónoma de México, Apartado Postal 20-364, Ciudad de México C.P. 01000, Mexico*

(Dated: March 14, 2024)

S1. Λ-SCHEME: THEORETICAL DETAILS

We consider the imaginary-time Green's function defined as $\mathcal{G}_{\alpha,\beta}(\tau) = -\langle T_\tau[\hat{\psi}_\alpha(\tau)\hat{\psi}^\dagger_\beta(0)]\rangle$, here the subindices α, β correspond to the left/right cavity photon and exciton, respectively. The Dyson's equation $\mathcal{G}^{-1}(z) = [\mathcal{G}^{(0)}(z)]^{-1} - \Sigma(z)$, is given in terms of the ideal $\mathcal{G}^{(0)}(z)$ Green's function which define a diagonal matrix

$$\mathcal{G}^{(0)}_{11}(z) = \frac{1}{z - \omega^L_c(\theta)}, \qquad \mathcal{G}^{(0)}_{22}(z) = \frac{1}{z - \omega^R_c(\theta)} \qquad \mathcal{G}^{(0)}_{33}(z) = \frac{1}{z - \omega_X}. \tag{1}$$

The self-energy gives the coupling between the left and right cavity photons $\Sigma_{12}(z) = \Sigma_{21}(z) = -t$, with t the tunneling coefficient. The light-matter coupling between the excitons and right cavity photons is given by $\Sigma_{23}(z) = \Sigma_{32}(z) = \Omega$.

In this case, as mentioned in the main text, the Green's function acquires an analytical form, in particular, the Green's function of the left cavity photons is given by

$$\mathcal{G}_{11}(z) = \frac{1}{z - \omega^L_c(\theta) - \frac{t^2}{z - \omega^R_c(\theta) - \frac{\Omega^2}{z - \omega_X}}}, \tag{2}$$

here, the Green's function develops three poles $\mathcal{G}^{-1}_{11}(\omega^i(\theta)) = 0$ which determine the energy $\omega^i(\theta)$ of the three quasi-particle branches i = LP,MP,UP. The Hopfield coefficients for the three-level scheme are given in terms of the quasi-particle residues of the branches The residue

$$Z_{li} = \left(\frac{\partial \mathrm{Re}[\mathcal{G}^{-1}_{ll}(\omega)]}{\partial \omega}\right)^{-1}\Bigg|_{\omega = \omega^i(\theta)}. \tag{3}$$

with $l = 1, 2, 3$. This gives the nine Hopfield coefficients that determine the composition of the three polariton branches in terms of the three bare states. This procedure can also be applied to conventional two-level polaritons [1] and agrees with the Hopfield coefficients obtained from direct diagonalization of the 2×2 Hamiltonian.

The features of the MP branch are obtained by following the pole of Eq. 1 around ω_X, in this case, we can approximate

$$\mathcal{G}_{11}(\omega_X + \omega) \approx \frac{1}{\omega_X + \omega - \omega^L_c(\theta) + \frac{t^2}{\Omega^2}\omega}, \tag{4}$$

this leads to a pole when

$$\omega(\theta) = \frac{[\omega^L_c(\theta) - \omega_X]}{1 + \left(\frac{t}{\Omega}\right)^2}, \tag{5}$$

* acamacho@fisica.unam.mx

† pirruccio@fisica.unam.mx

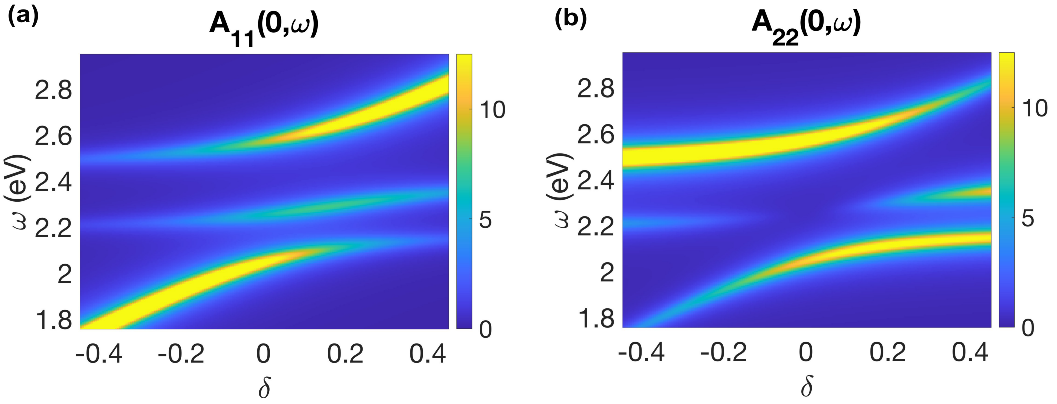

Supplementary Figure 1. Spectral function of the (a) Left and (b) Right cavity photons as a function of the left cavity detuning, $\delta = \delta_{LX}$. Around zero detuning we observe that the middle polariton decouples from the right cavity photon.

that is, the energy of the middle polariton is given by $\omega_{\mathrm{MP}}(\theta) = \omega_X + \frac{[\omega_c^L(\theta)-\omega_X]}{1+\left(\frac{t}{\Omega}\right)^2}$ as given in the main text. This defines an energy window of

$$\Delta\sigma = \frac{\Omega^2}{\sqrt{\left(\gamma_c^{(R)}\right)^2 + |\omega_X - \omega_c^R(\theta=0)|^2}},$$

in which the analogue of EIT holds.

The broadening of the photon and exciton lines can be included via additional terms which account for the losses. Then, the experimental model departs from the idealized scenario discussed before. The broadening of the right cavity photons reduces the energy window $\Delta\sigma$ while the broadening of the exciton lines leads to imperfect slow-light where the right cavity photons remains coupled to the middle polariton even at resonance $\omega_c^R(\theta=0) = \omega_X$. This coupling is small as long as $\Delta\sigma/\gamma_X \gg 1$. For our experimental conditions, we estimate the ratio

$$\Delta\sigma/\gamma_X \approx 10 - 50,$$

that is, we expect to see clear signatures of slow-light with right cavity photons effectively decoupled from the middle polaritons under resonant conditions.

In Fig. 1 we show the spectral function at normal incidence of the left (a) and right (b) cavity photons as a function of the detuning $\delta = \delta_{LX}$. The vanishing value of the spectral function $A_{22}(\mathbf{0},\omega)$ around zero detuning signals the full decoupling of the right cavity photon from the middle polariton.

The vanishing of the right cavity photon component on the middle polariton is equivalent to the transparency window observed in electromagnetically induced transparency. This implies that the possibility to observe the middle polariton depends on the arrangement of the stacked cavities. In particular, we observe the middle polariton in reflectance as light is injected from the left cavity. If light were injected from the right cavity, then the reflectance spectrum would not signal the presence of this branch. For the same reason, transmittance would not reveal the MP branch. In this sense, the middle polariton is dark under certain circumstances.

S2. TWO-LEVEL VS THREE-LEVEL POLARITONS

To understand the differences between our inter-cavity polaritons and the physics accessible with conventional two-level polaritons, in this section, we provide a direct comparison between these two kind of polaritons. For simplicity, we consider that the bare photonic mass is quadratic, with the same mass, m_c, for both two- and three-level polaritons.

Two-level polaritons: In conventional two-level polaritons, the energy of the polariton branches is given by

$$\epsilon^{(2)}_{\mathrm{UP/LP}}(\mathbf{k}) = \frac{\omega_X + (\omega_X + \delta + \frac{k^2}{2m_c}) \pm \sqrt{\left(\delta + \frac{k^2}{2m_c}\right)^2 + 4\Omega^2}}{2} \quad (6)$$

the polariton mass defined as

$$\frac{m^{(2)}_{\mathrm{UP/LP}}(\mathbf{k})}{m_c} = \frac{1}{\frac{d^2\epsilon^{(2)}_{\mathrm{UP/LP}}(\mathbf{k})}{dk^2}}, \tag{7}$$

for zero-momentum, this expression gives

$$\frac{m^{(2)}_{\mathrm{UP/LP}}(\mathbf{0})}{m_c} = \frac{2\sqrt{\delta^2+4\Omega^2}}{\pm\delta+\sqrt{\delta^2+4\Omega^2}}, \tag{8}$$

this remarks that the mass of the upper (lower) polariton can be increased by a large negative (positive) detuning. One should stress that once the detuning is fixed, there are no longer free parameters to tune and control the dispersion of the polaritons. In Fig. 2(a) we show the dispersion of the a heavy-mass upper polariton (purple line), while in Fig. 2 (b) and (c) we plot with green dots the Hopfield coefficients of this branch, which give the composition of the UP in terms of the bare exciton and photons.

Three-level polaritons.- In a Λ-scheme, the polariton mass on resonance, that is, when the energy of the MP lies on top of the exciton energy can be controlled by means of the ratio t/Ω, as discussed in the main text. Furthermore, the detuning of the right cavity with the exciton energy determines the angular width of the slow-light. To make the comparison fair with the two-level polaritons, we take a MP with the same balance of photonic-excitonic component. Since the MP becomes photonic at large angles, we take the UP for the two-level system to distinguish the differences and similitudes.

In Fig. 2 we compare the dispersion of the upper polariton of the two-level scheme with different middle polaritons of the three-level one, all sharing the same zero-momentum effective mass $m(\mathbf{0})$. For clarity in our comparison, we shift the upper polariton to zero energy, i.e., the blue dots correspond to the dispersion $\epsilon^{(2)}_{\mathrm{UP}}(\mathbf{k}) - \epsilon^{(2)}_{\mathrm{UP}}(\mathbf{0})$. In this case, we take an effective mass of $m/m_c = 4$. The red, yellow, and purple dots illustrate the degree of flexibility unveiled by the three-level scheme that allows us to produce MP states with a flatten dispersion which can exceed or be smaller than that of the two-level UP.

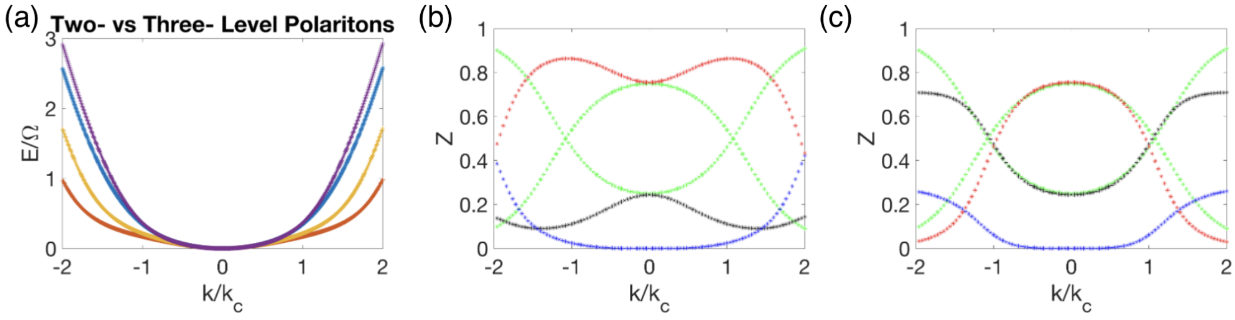

Supplementary Figure 2. (a) Comparison between the two-level and three-level polaritons as a function of the in-plane momentum in units of $k_c = \sqrt{2m_c\Omega}$. The blue dots correspond to the UP shifted to zero energy $\epsilon^{(2)}_{UP}(\mathbf{k}) - \epsilon^{(2)}_{UP}(\mathbf{0})$, while the red, yellow and purple dots correspond to different detunings of the right cavity with respect to the exciton mode $(\omega_X - \omega_c^R)/\Omega = 3, 1.5$, and -1.5 respectively. (b)-(c) Hopfield coefficients for the middle polariton of the three-level scheme following the same color code than in the main text. The green dots correspond to the Hopfield coefficients of the upper polariton of the two-level case shown in (a). (b) For $(\omega_X - \omega_c^R)/\Omega = 3$ the excitonic and photonic fractions of the MP of the three-level scheme strongly deviate from the excitonic and photonic components of the two-level upper polariton. (c) For $(\omega_X - \omega_c^R)/\Omega = -0.5$ the excitonic and (left) photonic fraction of the three-level scheme closely resemble those of the two-level upper polariton.

In Fig. 2(b) and (c), by using the same color code as in the main text, we show the generalized Hopfield coefficients for the three-level middle polariton corresponding to the red and purple dots of Fig. 2(a), respectively. The green dots are the Hopfield coefficients for the two-level upper polariton considered in Fig. 2(a). This shows that by only tweaking the right photon detuning the Hopfield coefficients for the Λ-scheme can strongly resemble or differ from the two-level polaritons with suppressed dispersion. Therefore, the three-level scheme can recover the dispersion of conventional two-level polaritons, but yields to a further control previously inaccessible.

Interestingly, even when the dispersion and Hopfield coefficients of the three-level middle polariton resemble the ones of the two-level polariton, the three-level polaritons retain their full inter-cavity nature, where excitons and photons are physically displaced.

S3. THE ROLE OF THE RIGHT CAVITY DETUNING

The quasiparticle properties of the pure intercavity polariton are robust against the value of the right cavity detuning $\delta_{RX} = \omega_c^R - \omega_X$. Indeed, its energy, mass and composition are completely independent of δ_{RX} and only depend on t and Ω. The pure intercavity polariton forms for $\delta_{LX} = 0$. Away from this condition, the MP acquires an intracavity component and starts depending on δ_{RX}.

To understand the role of δ_{RX} away from the pure intercavity condition, in Fig. 3 we show the energy and polariton mass for three values of δ_{RX}/eV: 0 (blue), 0.15 (red) and -0.15 (yellow).

In both panels of Fig. 3 it is evident that all the curves intersect at $\delta_{LX} = 0$, that is, the pure intercavity polariton is independent of δ_{RX}. Away from this condition, the curves depart and the dependence on δ_{RX} becomes notorious. As expected, with large detuning one can increase arbitrarily the mass of the polaritons. For $\delta_{RX}/\text{eV} = \pm 0.15$ we already see that the polariton mass increases significantly (red and yellow large dots). This is, however, at the expense of: a) breaking the pure intercavity polariton, i.e., the MP becomes mixture of inter-intra cavity polariton. b) The energy of the MP is pushed away from the bare exciton energy. c) The MP cedes its photonic component.

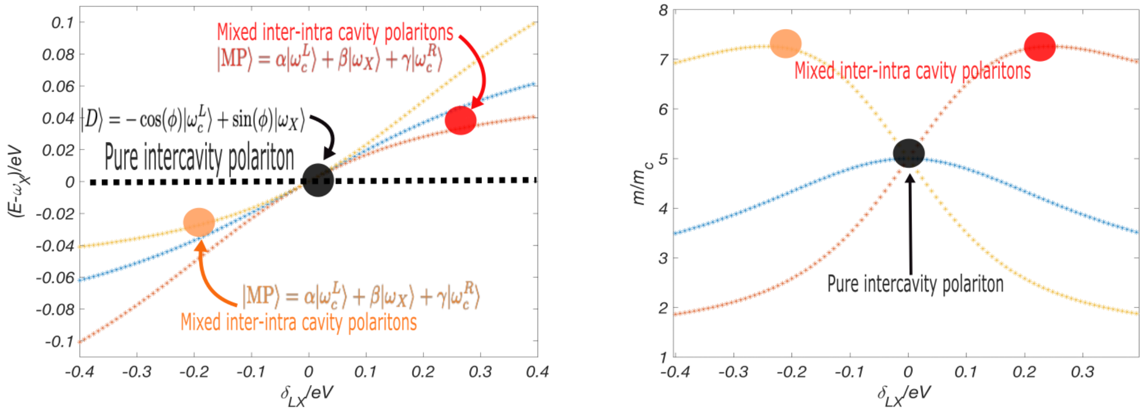

Supplementary Figure 3. Robustness of intercavity polariton to varying δ_{RX}. Quasiparticle properties of pure intercavity polaritons are independent of δ_{RX}. (a) Energy as a function of δ_{LX} for several values of δ_{RX}: the blue, yellow and red dots correspond to $\delta_{RX} = 0$, $\delta_{RX} = -0.15$ and $\delta_{RX} = 0.15$, respectively. (b) Middle polariton mass for the same values of δ_{RX} used in panel (a).

For the sake of comparison, in Fig. 3 we take $t/\text{eV} = 0.24$ and $2\Omega/\text{eV} = 0.24$, which are relevant values of the experimental cases (see Table 1). Note that, these parameters slightly vary from cavity to cavity.

One should note that while the quasiparticle properties of the pure intercavity polariton are completely independent of δ_{RX}, the angular range where the polaritons retain its intercavity nature, does depend on δ_{RX}, this range can be indeed controlled, as shown in the previous section. This emphasises, again, the versatility of the three-level scheme compared to two-level conventional polaritons.

S4. EMERGENCE OF HEAVY-MASS INTERCAVITY POLARITONS

We now show additional experimental data that further illustrate the emergence of an intercavity polariton with a tuneable dispersion. As explained in the main text, the polariton bands depend on the tunneling ratio, the Rabi splitting, and the different energy levels. We introduce the cavity detuning from the exciton energy at normal incidence $\delta_{LX} = \omega_c^{(L)}(\theta = 0) - \omega_X$ and $\delta_{RX} = \omega_c^{(R)}(\theta = 0) - \omega_X$.

Figures 4 (a)-(f) show the reflectance spectrum for varying detuning δ_{LX} with the remaining parameters as detailed in Table [1]. Our fitting allows to tune freely the cavity detunings, while the tunneling rate (t), the Rabi coupling (Ω), and the bare exciton energy (ω_X) remain reasonably fixed. From left to right we observe the emergence of a purely middle intercavity polariton formed by a left cavity photon and an exciton localized in the right cavity. This is demonstrated by the quasiparticle residues shown in the bottom of Figs. 4 (a)-(f).

Fig. 4	δ_{LX}[eV]	δ_{RX}[eV]	t[eV]	2Ω[eV]	ω_X[eV]
a	-0.37	0.12	0.21	0.26	2.23
b	-0.18	0.09	0.21	0.26	2.23
c	-0.08	0.13	0.21	0.26	2.23
d	-0.03	0.13	0.21	0.26	2.23
e	0.00	0.17	0.23	0.245	2.25
f	0.07	0.15	0.21	0.26	2.23

Supplementary Table 1. Theoretical parameters of the polariton branches in Fig. 4.

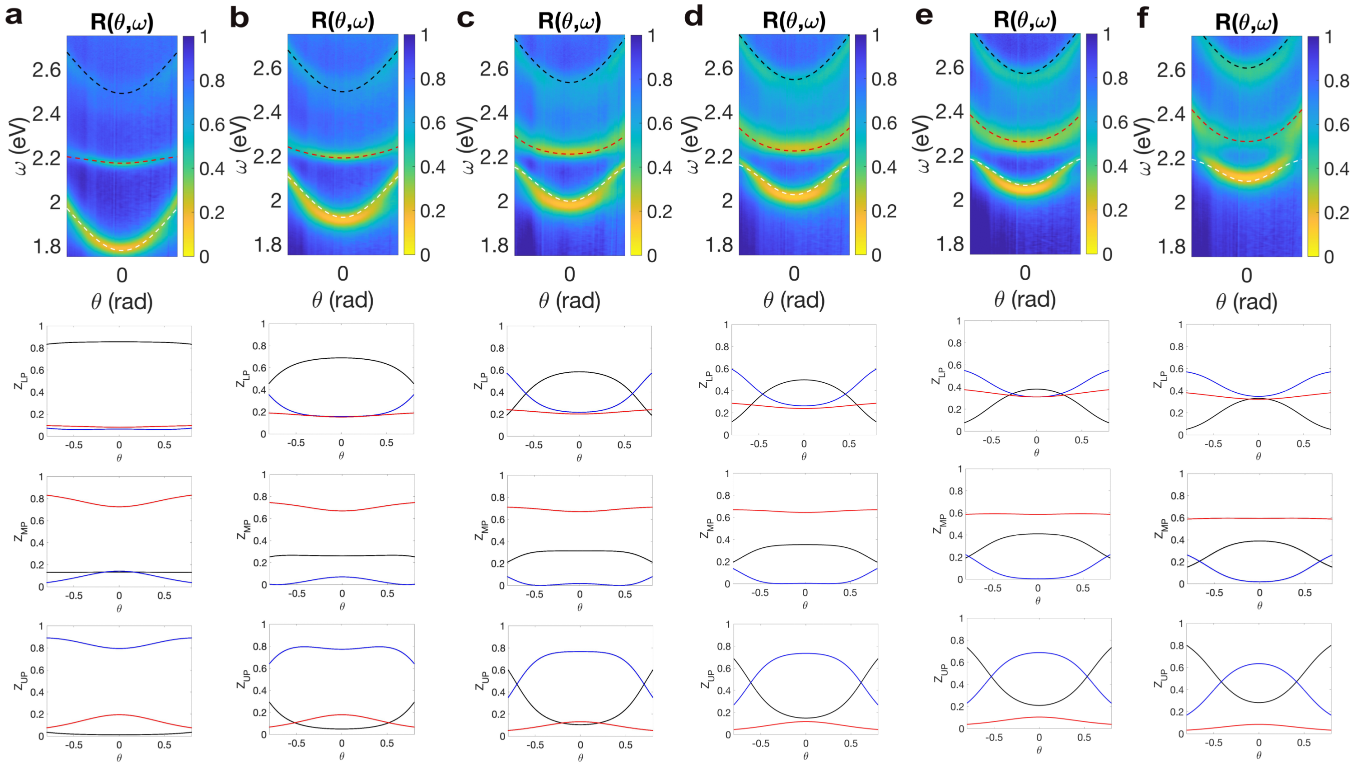

Supplementary Figure 4. s-polarized reflectance as a function of the incident angle and the energy for several cavity detunings. The dashed curves represent the energies of the three polariton branches, obtained with the parameters given in Table [1]. In the bootom row, we show the quasiparticle residues, i.e., the Hopfield coefficients, of the three quasiparticle branches following the color code of the main text.

The evolution of the MP Hopfield coefficients illustrate that, as the left cavity detuning is driven to $\delta_{LX} = 0$, the right cavity photon decouples from the MP causing the suppression of its dispersion. For $\delta_{LX} = 0$, although the

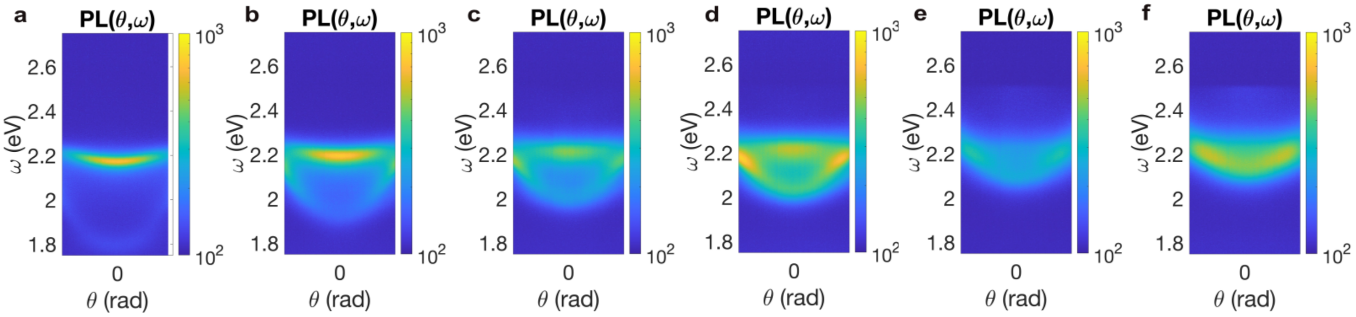

Supplementary Figure 5. s-polarized fluorescence for the same parameters as in Fig. 4 and Table 1. From left to right we observe a strong suppression of the MP fluorescence as the left cavity detuning approaches zero.

polariton energy matches the bare exciton, the MP does not lose its photonic character because it retains a significant residue of the left cavity photon.

In Fig. 5 we show the evolution of the fluorescence as a function of the detuning. Pumping the system at $\omega_p = 2.62$eV ensures that for $\delta = \delta_{LX} = 0$ we drive coherently the UP which consists primarily of right cavity photons. The absence of the left photonic component in the driven UP strongly suppresses the emission of the MP for $\delta = \delta_{LX} = 0$

We should note that the right cavity photons are the dominant component of the UP in all of our cavity detunings. However, as we drive at $\omega_p = 2.62$eV, for large negative detuning, this driving is off-resonant, that is, it lies far above the energy of the upper polariton, and thus, photons are injected in left and right cavities. Therefore, for negative detunings, we observe emission from both the middle and lower polariton branches.

S5. POLARITON DYNAMICS

In the main text we focus on the short-time dynamics of the polaritons. Here, we provide the experimental data in an extended time window. Figure 6 shows the LP (a) and MP (b) fluorescence decay for $\delta_{LX}/\text{eV} = -0.37$ (blue), on resonance $\delta_{LX}/\text{eV} = 0$ (purple) and $\delta_{LX}/\text{eV} = 0.20$ (green). The shaded pink area corresponds to the time window discussed in the main text where we focused on the initial decay. As a consequence of the coupling to the triplet state by intersystem crossing for large negative detunings, or to the bare exciton states for positive detunings, the decay of the LP for $t > 10$ns after the laser pulse arrival becomes more complex. In the former case, where the energy of the LP approaches the triplet state, we see the presence of a long-living state which does not decay entirely in the considered time window. A similar effect, although less pronounced, is seen as the LP shifts toward the bare exciton energy. An intermediate situation is observed for detunings around $\delta_{LX}/\text{eV} = -0.2$. The ability to control the dynamics of the LP was extensively discussed for a single cavity [2].

On the other hand, the MP energy lies very far away from the triplet state, thus the triplet state does not participate in the MP dynamics and its decay is given by the slow-light physics discussed in the main text. For $t > 10$ns after the laser pulse arrival, the MP time evolution resembles the dynamics of the ErB/PVA thin film in the absence of the cavity. This can be appreciated by comparing the black curve in Fig. 6(b) with the MP decays.

In Fig. 6(b) we show with red curve the IRF obtained by measuring a 0.5 M solution of ErB in water which is known to decay mono-exponentially with a lifetime well below the time resolution of our instrument. This choice ensures that the detector wavelength-dependent response does not play a role in our measurements. The same experimental conditions as for the cavity measurements are used. The full-width at half-maximum of this decay is approximately 350 ps. Having characterized the IRF curve, the short-time fit of the LP and MP decays can be safely conduct by excluding the first 600 ps after the intensity maximum. This time value is labelled as $t = 0$ ns in Fig.3(a) of our manuscript. It is important to highlight that within the shaded pink area, i.e., within the first 10 ns from the arrival of the laser pulse, the dynamics of the ErB molecule in absence of the cavity can be excellently modeled by a single exponential. We find that $A = 0.9$, $\Gamma = 4.9$ ns^{-1} and $\eta = 0.24$ ns^{-1}. Thus the rich interplay observed for the MP and its overall slow dynamics cannot be accounted for with the ErB photophysics.

Our proposal reveals a competition between the dispersion of the polariton bands and the accessible decay channels. On the one hand, the reduction of the middle polariton curvature increases the number of states lying at the exciton reservoir energy. That is, the flattening of the MP is related to the expected accelerated decay. On the other hand,

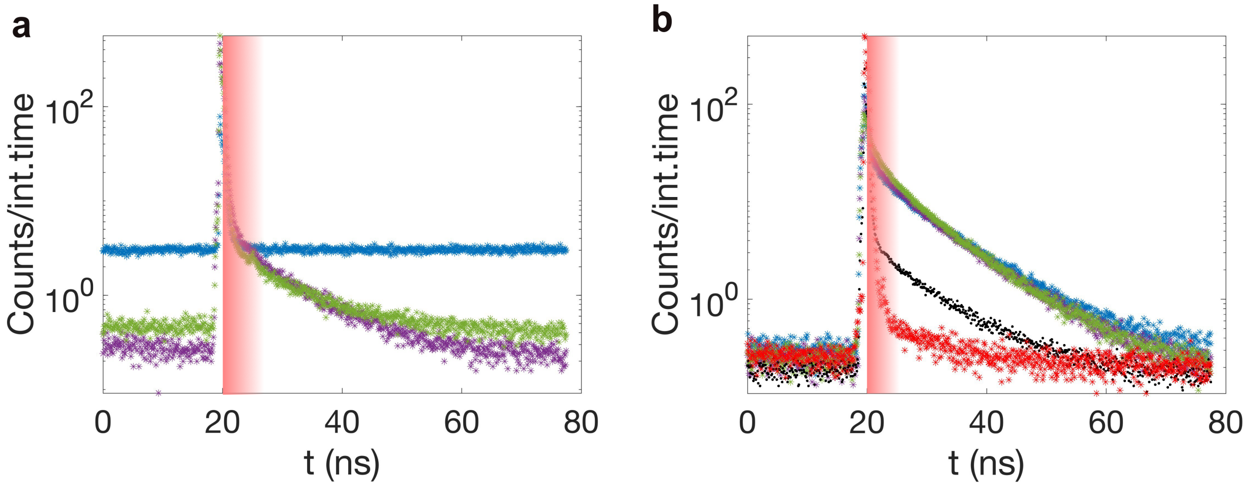

Supplementary Figure 6. Fluorescence decays of the LP (a) and MP (b) for different cavity detunings: $\delta_{LX}/\mathrm{eV} = -0.37$ (blue), on resonance $\delta_{LX}/\mathrm{eV} = 0.00$ (purple) and $\delta_{LX}/\mathrm{eV} = 0.20$ (green). In (b), the black curve represents the decay of the ErB/PVA thin film in the absence of the cavity, while the red curve corresponds to the IRF. The shaded pink area corresponds to the time window discussed in the main text.

the strategy we employed to flatten the band, i.e., the Λ scheme, is directly accompanied by a reduction of the right cavity photon component, that prevents direct decay of the exciton into a right cavity photon.

[1] Yesenia A García Jomaso, Brenda Vargas, David Ley Dominguez, César L Ordoñez-Romero, Hugo A Lara-García, Arturo Camacho-Guardian, and Giuseppe Pirruccio, "Fate of the upper polariton: Breakdown of the quasiparticle picture in the continuum," Physical Review B **107**, L081302 (2023).

[2] Kati Stranius, Manuel Hertzog, and Karl Börjesson, "Selective manipulation of electronically excited states through strong light–matter interactions," Nature Communications **9**, 2273 (2018).
